# Impact of saline sprouting on antioxidant properties and bioactive compounds in chia seeds

**DOI:** 10.1515/biol-2025-1125

**Published:** 2025-07-24

**Authors:** Dani Dordevic, Jana Hrachovska, Simona Dordevic, Ivan Kushkevych

**Affiliations:** Department of Plant Origin Food Sciences, Faculty of Veterinary Hygiene and Ecology, University of Veterinary Sciences Brno, Palackeho tr. 1946/1, 612 42, Brno, Czech Republic; Department of Experimental Biology, Faculty of Science, Masaryk University, 62500, Brno, Czech Republic

**Keywords:** salt stress, seawater, germination, antioxidant capacity

## Abstract

The consumption of chia seeds has surged in recent years, primarily due to their beneficial chemical composition and health effects. Sprouting chia seeds can enhance the content of essential nutrients, including antioxidants and vitamins. The study investigates the impact of sprouting on the bioactive compounds and antioxidant activity of chia seeds under both favorable and stressful conditions. Chia seeds were sprouted in tap water, distilled water, and varying concentrations of seawater. The parameters analyzed included antioxidant activity, the reducing capacity of antioxidants, total polyphenol content, flavonoid content, and phenolic profile. Results indicated that sprouting significantly influences antioxidant activity in seeds sprouted in tap and distilled water, with a decrease observed only in the 2,2′-azinobis(3-ethyl-2,3-dihydrobenzothiazol-6-sulfonate) method for distilled water. Additionally, sprouting in both water types led to a statistically significant increase (*p* < 0.05) in reducing capacity and total polyphenol content. Under high salinity conditions, sprouting in 100% seawater resulted in a significant increase (*p* < 0.05) in antioxidant activity, reducing capacity, and total polyphenol content. These findings suggest that sprouting chia seeds, particularly under saline conditions, could enhance their nutritional profile, presenting potential applications in the food and nutrition industry and indicating possibilities for ecological cultivation.

## 1 Introduction

*Salvia hispanica* L., commonly known as chia, is an annual herb belonging to the order Lamiales, family Lamiaceae, and the genus *Salvia* [1,2,3]. Chia is a tropical to subtropical plant that blooms in the summer and is highly sensitive to photoperiod, requiring specific light conditions for optimal growth [3,4,5]. Originating in Mexico and Guatemala, the genus *Salvia* encompasses approximately 900 species, and while initially confined to these regions, chia has since spread globally. Mexico remains the largest producer of chia seeds today [4,6].

Chia seeds have been utilized as a food source since 3,500 BCE, gaining significant importance between 1,500 and 900 BCE due to their nutritional and therapeutic potential [7]. Chia seeds are small, flat, and oval, typically 1.9–2 mm in length, 1–1.5 mm in width, and 0.8–1 mm in thickness. They have a smooth, shiny surface and are generally tasteless and odorless [4,7,8,9]. Chia seeds are rich in antioxidants, preventing the autooxidation of fatty acids and allowing for long-term storage [7]. They can be used in various forms in the food industry, including whole seeds, ground seeds, chia flour, chia oil, chia gel, and sprouts [1,4,9].

Germination is a crucial stage where seeds begin metabolic activity, spurred by hormones like gibberellins and abscisic acid, initiating embryo growth into seedlings [10,11]. Differentiating between hypogeal and epigeal germination in angiosperms reveals how seeds either keep cotyledons underground, utilizing stored nutrients until their depletion, or emerge above ground as photosynthetic organs [12,13]. Essential environmental factors such as water, temperature, oxygen, and light influence seed germination, ensuring optimal conditions for the absorption of water, enzymatic activation, and subsequent growth. During germination, seeds rely on stored nutrients like starch, proteins, and fats, which undergo enzymatic breakdown to provide energy and materials for initial growth before transitioning to autotrophic nutrition [10,13]. Understanding these processes sheds light on how seeds adapt to their environments, utilizing diverse strategies to ensure successful germination and early seedling development across different plant species [12,14].

Otherwise, soil salinity and sodicity present significant challenges to agriculture globally, making salt tolerance in crops a crucial trait and a key area of research. High salinity negatively impacts crops in various ways, including inducing drought stress, ion toxicity, nutrient imbalances, oxidative stress, disruption of metabolic functions, membrane instability, and decreased cell division and growth. Given the growing scarcity of freshwater resources, it is increasingly important for the environment and sustainable agriculture to explore the use of seawater or saline water in food production, as this could help mitigate freshwater shortages and allow cultivation in saline-affected areas [15,16].

The aim of the study was to investigate the impact of saline sprouting on the antioxidant properties and bioactive compounds of chia seeds. This investigation aims to provide insights into how sprouting chia seeds in saline environments can potentially enhance their antioxidant value, thereby suggesting applications in the food and nutrition industry and implications for sustainable cultivation practices.

## 2 Materials and methods

**Figure 1:**
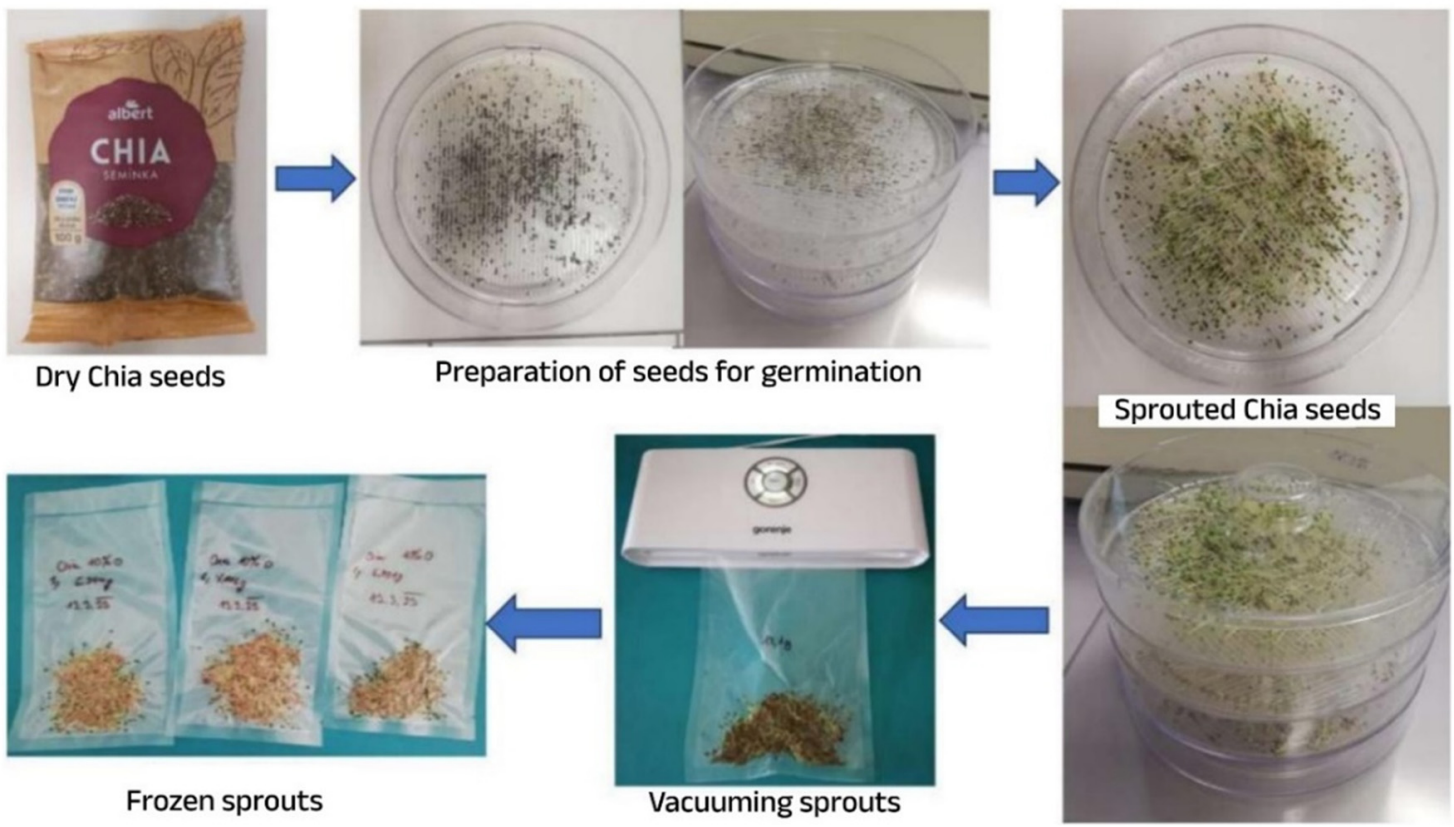
The process of sample preparation.

For germination, Multiflora s.r.o. germination containers with a diameter of 200 mm were used. The samples for germination consisted of chia seeds (*Salvia hispanica* L.) purchased from Albert Česká republika, s.r.o. Seeds weighing 2 g were placed into each germination container, followed by the addition of 10 mL of tap water, distilled water, seawater, or a solution of distilled and seawater. The concentrations of the distilled and seawater solutions were 5, 10, 20, 50, 60, 70, 80, 90, and 100%. The amounts of tap water, distilled water, and seawater used for each sample are detailed in Table 1. The seawater was collected in Poland, near the city of Gdańsk, at coordinates 54.3559544N, 18.8331400E. Before use, the seawater was filtered through filter paper. Seeds were allowed to germinate for 7 days at laboratory temperature under light conditions but away from direct sunlight [17]. After 7 days, the sprouts were harvested, weighed, vacuum-packed, and frozen. The whole process of germination is shown in Figure 1.

**Table 1:** Amount of tap water, distilled water, and seawater used for each sample

Sample	Amount of distilled, tap, and seawater used
Dry chia seeds	Water conditions
Germination with 5% seawater	0.5 mL seawater + 9.5 mL distilled water
Germination with 10% seawater	1 mL seawater + 9 mL distilled water
Germination with 20% seawater	2 mL seawater + 8 mL distilled water
Germination with 50% seawater	5 mL seawater + 5 mL distilled water
Germination with 60% seawater	6 mL seawater + 4 mL distilled water
Germination with 70% seawater	7 mL seawater + 3 mL distilled water
Germination with 80% seawater	8 mL seawater + 2 mL distilled water
Germination with 90% seawater	9 mL seawater + 1 mL distilled water
Germination with 100% seawater	10 mL seawater
Germination with distilled water	10 mL distilled water
Germination with tap water	10 mL tap water

For each sample, the following parameters were determined: antioxidant activity using the ferric reducing antioxidant potential (FRAP) method, 1,1-diphenyl-2-picrylhydrazyl (DPPH) method, 2,2′-azinobis(3-ethyl-2,3-dihydrobenzothiazol-6-sulfonate) (ABTS) method, cupric reducing antioxidant capacity (CUPRAC) method, flavonoid content, and the total polyphenol content using the Folin–Ciocalteu method.

An extract was prepared from each sample, which was then used to determine each parameter. Each analysis was measured six times for each sample.

### 2.1 Preparation of extracts

The sample was weighed (0.1 g) into an Erlenmeyer flask, and 20 mL of an ethanol solution was added. The ethanol solution was prepared from 96% ethanol and distilled water in a 1:1 ratio. The flasks were submerged in an ultrasonic bath (Radiotechnika, Czech Republic, RS 2T), and the samples were extracted for 30 min. After extraction, the flasks were cooled, and their contents were filtered using syringe filters (Filtratech, nylon syringe filter 0.45 µm). The prepared extracts were then used for all analyses [18].

### 2.2 FRAP

The FRAP method measures antioxidant activity based on the ability of antioxidants to reduce ferric ions in a redox reaction. Antioxidants reduce ferric ions in the colorless 2,4,6-tripyridyl-*S*-triazine (TPTZ) complex, forming a blue ferrous complex, with absorbance at 593 nm indicating antioxidant levels. The method used was a slight modification of Behbahani et al. [19].

To determine antioxidant activity, the following reagents were prepared: TPTZ solution, FeCl_3_·6H_2_O solution, acetate buffer, working solution, and Trolox solution (TPTZ solution: 0.0312 g TPTZ in 10 mL diluted HCl, sonicated for 8 min; FeCl_3_·6H_2_O solution: 0.032 g FeCl_3_ in 10 mL distilled water, sonicated for 8 min; acetate buffer: 1.55 g NaCH_3_COO·3H_2_O in 8 mL CH_3_COOH, diluted to 500 mL, pH 3.6; working solution: 50 mL acetate buffer, 5 mL TPTZ solution, 5 mL FeCl_3_ solution; and Trolox solution: 12.5 mg Trolox in 10 mL ethanol). Samples were prepared in dark vials by mixing 180 µL sample extract, 300 µL water, and 3.6 mL working solution, and incubated for 8 min. A blank was prepared with 960 µL of water and 7.2 mL of working solution and also incubated for 8 min. After incubation, sample absorbance was measured at 593 nm using the blank to zero the spectrophotometer. Results were expressed in µg/mL Trolox equivalent. Concentrations of Trolox in mmol/L were the following: 0, 0.0375, 0.1, 0.2, 0.4, 0.8, 1.125, and 1.6. The regression was 0.9987.

### 2.3 DPPH

The DPPH method assesses antioxidant activity by measuring the reaction between antioxidants in the sample and the stable radical DPPH. Antioxidants reduce the purple DPPH radical to a colorless DPPH-H molecule. This color change is measured at 517 nm and indicates antioxidant activity.

The procedure begins with the preparation of the DPPH solution by dissolving 0.0039 g of DPPH in 100 mL of ethanol and protecting the solution from light by wrapping the flask in aluminum foil. Next, a control solution is prepared by mixing 3 mL of ethanol with 1 mL of 0.1 mM DPPH solution in a dark vial, vortexing the solution, and incubating it in the dark for 30 min. For sample preparation, 3 mL of the sample extract is mixed with 1 mL of 0.1 mM DPPH solution in a dark vial, vortexed, and incubated in the dark for 30 min. After the incubation period, the absorbance is measured at 517 nm using ethanol as the blank. The absorbance of both the sample and the control solution is recorded [20,21]. The calculation was done according to the following formula (Abs = absorption):

\[\text{DPPH}\hspace{.25em}( \% )={[}({\text{Abs}}_{\text{DPPH}}-{\text{Abs}}_{\text{DPPH}})/{\text{Abs}}_{\text{DPPH}}]\times 100.]\]

### 2.4 ABTS

The ABTS method measures the ability of antioxidants to neutralize free radicals. Antioxidants act as hydrogen donors, quenching the ABTS radical cation, resulting in a color change that corresponds to a change in absorbance. This change is spectrophotometrically measured at a wavelength of 735 nm. First, the ABTS solution and potassium persulfate solution were prepared. The ABTS solution was made by dissolving 0.0384 g of ABTS in 10 mL of distilled water. The potassium persulfate solution was prepared by dissolving 0.0662 g of potassium persulfate in 100 mL of distilled water. The reaction solution, which needs to be prepared 12–16 h in advance, was made by mixing 10 mL of the ABTS solution with 10 mL of the potassium persulfate solution and storing it at room temperature in the dark until use. After incubation, the reaction solution was diluted to achieve an absorbance of 0.7 at 735 nm, by mixing approximately 1.37 mL of the ABTS solution with 70 mL of ethanol. This dilution was adjusted until the correct absorbance was reached. For the spectrophotometric measurement, 20 µL of the sample extract was mixed with 1,980 µL of the diluted reaction solution in 10 mL test tubes. Each sample was mixed with the reaction solution twice and incubated in the dark for 5 min. The spectrophotometer was zeroed using 96% ethanol, and the absorbance of the samples was measured at 735 nm. The absorbance of a control solution, prepared by mixing 96% ethanol with distilled water in a 1:1 ratio, was also measured [22]. The calculation was done according to the following formula (Abs = absorption):

\[\text{ABTS}\hspace{.25em}( \% )={[}({\text{Abs}}_{\text{ABTS}}-{\text{Abs}}_{\text{sample}})/{\text{Abs}}_{\text{ABTS}}]\times 100.]\]

### 2.5 CUPRAC

The CUPRAC method tests the reduction of copper complexes by antioxidants in the sample, similar to the FRAP method. Antioxidants reduce cupric complexes to the cuprous form, causing a change in absorbance measured at 450 nm; higher absorbance indicates greater reducing capacity [23]. The procedure follows a slightly modified version of Özyürek et al. [24]. First, reagents were prepared: cupric chloride solution, NH_4_Ac buffer, and neocuproine solution. The cupric chloride solution was made by dissolving 0.4626 g of CuCl_2_·2H_2_O in 250 mL of distilled water. The NH_4_Ac buffer was prepared by dissolving 19.2 g of ammonium acetate in 250 mL of distilled water. The neocuproine solution was prepared by dissolving 0.0390 g of neocuproine (2,9-dimethyl-1,10-phenanthroline) in 25 mL of 99% ethanol. A blank sample was prepared in a 10 mL test tube by pipetting 2 mL of cupric chloride solution, 2 mL of neocuproine solution, 2 mL of buffer, and 2.2 mL of solvent (a 1:1 mixture of 96% ethanol and distilled water). The blank sample was incubated in the dark for 1 h. For sample preparation, 1 mL of the sample extract was pipetted into a 10 mL test tube, followed by 1 mL each of cupric chloride solution, neocuproine solution, and buffer, and 0.1 mL of solvent. The test tubes were incubated in the dark for 1 h. After incubation, the absorbance of the samples was measured spectrophotometrically against the blank sample at 450 nm. Concentrations of Trolox in mmol/L were the following: 0, 0.0375, 0.1, 0.2, 0.4, 0.8, 1.125, and 1.6. The regression was 0.9987.

### 2.6 Determination of total polyphenol content by the Folin–Ciocalteu method

The Folin–Ciocalteu reagent, a mixture of tungsten and molybdenum complexes, reacts with polyphenols in the sample, reducing these ions and forming blue-colored products. The total polyphenol content is determined by measuring the absorbance of these blue products at 765 nm. The procedure follows a slightly modified version of Tomadoni et al. [25].

A blank sample was prepared in a 25 mL volumetric flask by adding 1 mL of distilled water, 4 mL of Na_2_CO_3_ solution (75 g/L), and 5 mL of Folin–Ciocalteu reagent (diluted 1:10 with distilled water). This mixture was incubated in the dark for 30 min. After incubation, the volumetric flask was topped up to the mark with distilled water immediately before measurement. For sample preparation, 1 mL of the sample extract was mixed with 5 mL of Folin–Ciocalteu reagent and 4 mL of Na_2_CO_3_ solution in a 25 mL volumetric flask. The Folin–Ciocalteu reagent and Na_2_CO_3_ solution were prepared in the same manner as for the blank sample. The prepared samples were incubated in the dark for 30 min, and after incubation, the volumetric flasks were filled to the mark with distilled water. Absorbance was then measured using a spectrophotometer, with measurements taken against the blank sample at a wavelength of 765 nm. Concentrations of gallic acid in mg/L were the following: 0.0125, 0.025, 0.05, 0.1, 0.2, and 0.5. The measures regression was 0.9986.

### 2.7 Statistical analysis

The values obtained from the analyses were statistically evaluated using IBM SPSS Statistics Substriction software. The “one-way analysis of variance” method was used for the analysis. Homogeneity of results was assessed using Levene’s test to determine if there was a statistically significant difference (*p* < 0.05). If the value was less than 0.05 (*p* < 0.05), the non-parametric Games–Howell test was used; if the value was greater than 0.05 (*p* > 0.05), the parametric Tukey test was applied. The results of the statistical evaluation are expressed as the mean ± standard deviation.

## 3 Results and discussion

The measured values of antioxidant activity using the FRAP method are presented in Table 2.

**Table 2:** Results of antioxidant activity determination of samples using the FRAP method

Samples	FRAP (µmol Troloxu/g)
Dry chia seeds	2.37 ± 0.74^a^
Germination with 5% seawater	18.04 ± 0.94^c^
Germination with 10% seawater	17.19 ± 0.85^c^
Germination with 20% seawater	16.63 ± 1.13^c^
Germination with 50% seawater	12.22 ± 0.41^d^
Germination with 60% seawater	10.67 ± 0.44^e^
Germination with 70% seawater	13.85 ± 0.17^f^
Germination with 80% seawater	17.48 ± 2.78^cbdf^
Germination with 90% seawater	16.62 ± 1.84^cf^
Germination with 100% seawater	22.63 ± 0.93^b^
Germination with distilled water	19.51 ± 2.27^cb^
Germination with tap water	18.56 ± 0.80^c^

Different letters in superscript indicate statistically significant differences (*p* < 0.05) between rows.

From the obtained data, it is evident that the antioxidant activity significantly increased during germination. The enhanced antioxidant activity is attributed to metabolic changes occurring during germination [26]. This primarily involves an increase in the concentration of antioxidant compounds, such as vitamin C, phenolic compounds, and flavonoids [27]. The increase in antioxidant activity may also be attributed to enhanced availability of existing phenolic compounds [28].

Germination with 5, 10, 20, 80, and 90% seawater, as well as with distilled and tap water, did not show statistically significant differences (*p* > 0.05) in antioxidant activity. In contrast, germination with 50, 60, and 70% seawater exhibited statistically significant differences (*p* < 0.05) compared to the other samples, showing the lowest antioxidant activities.

Chia germinated in 100% seawater exhibited the highest antioxidant activity. This could be attributed to biochemical changes induced when plants are exposed to stressful conditions. Excessive salt exposure leads to the production of reactive oxygen species, which can severely disrupt plant cells and their metabolism [29]. To mitigate the harmful effects of reactive oxygen species, plants have developed complex antioxidant systems [30]. By enhancing antioxidant capacity, plants are able to tolerate higher concentrations of salt [31].

The results of antioxidant activity determined by the DPPH method are presented in Table 3.

**Table 3:** Results of antioxidant activity determination of samples by the DPPH method

Samples	DPPH (%)
Dry chia seeds	0.00^a^
Germination with 5% seawater	50.99 ± 2.34^bfh^
Germination with 10% seawater	47.08 ± 1.73^bdf^
Germination with 20% seawater	50.11 ± 1.43^bh^
Germination with 50% seawater	38.02 ± 1.72^ce^
Germination with 60% seawater	35.41 ± 2.18^c^
Germination with 70% seawater	41.75 ± 3.09^deg^
Germination with 80% seawater	45.90 ± 1.40^fg^
Germination with 90% seawater	51.63 ± 1.39^h^
Germination with 100% seawater	69.53 ± 0.74^i^
Germination with distilled water	61.69 ± 0.39^j^
Germination with tap water	48.24 ± 2.16^bgh^

Different letters in superscript indicate statistically significant differences (*p* < 0.05) between rows.

The antioxidant activity of non-germinated chia seeds was 0.00%, but it increased significantly during germination. The difference in antioxidant activity between non-germinated chia seeds and chia seeds after 7 days of germination was statistically significant (*p* < 0.05). Similar results were reported by Abdel-Aty et al. [28], where the antioxidant activity of chia seeds germinated in distilled water increased tenfold after 7 days of germination. According to Beltrán-Orozco et al. [3], the increase in antioxidant activity during germination is associated with higher levels of flavonoids and ascorbic acid.

The highest antioxidant activity was achieved in chia seeds germinated in 100% seawater. These germinated chia seeds showed a statistically significant difference (*p* < 0.05) compared to all other samples. The results of determining antioxidant activity using the DPPH method are presented in Table 4.

**Table 4:** Results of determining the antioxidant activity of samples using the DPPH method

Samples	DPPH (%)
Dry chia seeds	0.00^a^
Germination with 5% seawater	50.99 ± 2.34^bfh^
Germination with 10% seawater	47.08 ± 1.73^bdf^
Germination with 20% seawater	50.11 ± 1.43^bh^
Germination with 50% seawater	38.02 ± 1.72^ce^
Germination with 60% seawater	35.41 ± 2.18^c^
Germination with 70% seawater	41.75 ± 3.09^deg^
Germination with 80% seawater	45.90 ± 1.40^fg^
Germination with 90% seawater	51.63 ± 1.39^h^
Germination with 100% seawater	69.53 ± 0.74^i^
Germination with distilled water	61.69 ± 0.39^j^
Germination with tap water	48.24 ± 2.16^bgh^

Different letters in superscript indicate statistically significant differences (*p* < 0.05) between rows.

The antioxidant activity of unsprouted chia seeds was 0.00%, but it increased during sprouting. The difference between the antioxidant activity of unsprouted chia seeds and chia seeds after 7 days of sprouting was statistically significant (*p* < 0.05). Similar results were achieved in a study by Abdel-Aty et al. [28], where the antioxidant activity of chia seeds sprouted in distilled water increased tenfold after 7 days of sprouting. According to Beltrán-Orozco et al. [3], the increase in antioxidant activity during sprouting is related to the increase in the content of flavonoids and ascorbic acid. The highest antioxidant activity was achieved in chia seeds sprouted in 100% seawater. These sprouted chia seeds showed a statistically significant difference (*p* < 0.05) from all other samples. The results of determining antioxidant activity using the ABTS method are presented in Table 5.

**Table 5:** Results of determining the antioxidant activity of samples using the ABTS method

Samples	ABTS (%)
Dry chia seeds	0.28 ± 0.13a^a^
Germination with 5% seawater	1.21 ± 0.12^be^
Germination with 10% seawater	1.16 ± 0.09^bde^
Germination with 20% seawater	1.06 ± 0.09^bde^
Germination with 50% seawater	1.14 ± 0.12^bde^
Germination with 60% seawater	0.93 ± 0.56
Germination with 70% seawater	0.72 ± 0.11^c^
Germination with 80% seawater	0.89 ± 0.13^cd^
Germination with 90% seawater	0.88 ± 0.16^bcd^
Germination with 100% seawater	0.64 ± 0.11^c^
Germination with distilled water	0.00 ± 0.00^a^
Germination with tap water	1.38 ± 0.18^e^

Different letters in superscript indicate statistically significant differences (*p* < 0.05) between rows.

During sprouting, there was an increase in antioxidant activity, with a statistically significant difference (*p* < 0.05) between the antioxidant activity of unsprouted chia seeds and chia seeds sprouted for 7 days. According to Salgado et al. [27], these results indicate the ability of chia sprouts to neutralize free radicals. The exception was chia seeds sprouted in distilled water, where the antioxidant activity was lower than that of unsprouted chia seeds, and the difference was statistically insignificant (*p* > 0.05).

The increase in antioxidant activity is a result of the accumulation of antioxidants that naturally occurs during sprouting [32]. The concentrations of various antioxidant substances increase during sprouting, which affects the resulting antioxidant activity. Pajak et al. [33] observed a statistically significant positive correlation between antioxidant activity determined by the ABTS method and the total polyphenol content, which increased during sprouting. Additionally, a lower but still statistically significant positive correlation was observed between antioxidant activity determined by the ABTS method and flavonoid content [33]. The results of determining the reducing power of antioxidants using the CUPRAC method are presented in Table 6. There was a several-fold increase in the reducing power of antioxidants during sprouting.

**Table 6:** Results of determining the reducing power of antioxidants using the CUPRAC method

Samples	CUPRAC (µmol Troloxu/g)
Dry chia seeds	1.34 ± 0.22^a^
Germination with 5% seawater	6.53 ± 0.42^cdej^
Germination with 10% seawater	6.62 ± 0.40^dj^
Germination with 20% seawater	6.63 ± 0.36^cdj^
Germination with 50% seawater	5.65 ± 0.31^ei^
Germination with 60% seawater	4.45 ± 0.11^f^
Germination with 70% seawater	4.81 ± 0.05^g^
Germination with 80% seawater	5.64 ± 0.19^h^
Germination with 90% seawater	6.21 ± 0.15^cdi^
Germination with 100% seawater	6.96 ± 0.08^j^
Germination with distilled water	7.66 ± 0.18^k^
Germination with tap water	6.44 ± 0.03^bcd^

Different letters in superscript indicate statistically significant differences (*p* < 0.05) between rows.

Many studies have described an increase in the reducing power of antioxidants during sprouting in various types of seeds. Examples include black bean seeds (*Phaseolus vulgaris* L.), wheat (Emmer and Einkorn) [34], and oat (*Avena sativa* L.) [34,35,36]. Khang et al. [35] hypothesized that the reducing activity of antioxidants is directly proportional to phenolic compounds, whose content increases during sprouting.

Samples germinated with 5% seawater did not show a statistically significant difference (*p* > 0.05) from samples germinated with 10, 20, 50, 90, and 100% seawater and from samples germinated with tap water. Germination with 60, 70, and 80% seawater and germination with distilled water showed statistically significant differences (*p* < 0.05) from all other samples and from each other. The results of determining the total polyphenol content using the Folin–Ciocalteu method are presented in Table 7.

**Table 7:** Results of determining the total polyphenol content using the Folin–Ciocalteu method

Samples	Total polyphenol content (mg gallic acid/g)
Dry chia seeds	0.01 ± 0.00^a^
Germination with 5% seawater	0.06 ± 0.00^b^
Germination with 10% seawater	0.06 ± 0.00^c^
Germination with 20% seawater	0.07 ± 0.00^bf^
Germination with 50% seawater	0.06 ± 0.00^bc^
Germination with 60% seawater	0.05 ± 0.00^d^
Germination with 70% seawater	0.05 ± 0.00^e^
Germination with 80% seawater	0.06 ± 0.00^bc^
Germination with 90% seawater	0.07 ± 0.00^f^
Germination with 100% seawater	0.08 ± 0.00^g^
Germination with distilled water	0.06 ± 0.00^bc^
Germination with tap water	0.06 ± 0.00^bc^

Different letters in superscript indicate statistically significant differences (*p* < 0.05) between rows.

From the resulting data, it is evident that there was an increase in total polyphenol content during germination, with the difference in total polyphenol content between ungerminated chia seeds and germinated chia seeds being statistically significant (*p* < 0.05). Similar results were obtained in studies that focused on the germination of various legumes, sunflower seeds (*Helianthus*), and radish seeds (*Raphanus sativus*) [33].

The increase in total polyphenol content is a metabolic change that occurs during seed germination, primarily due to the increased activity of endogenous enzymes. In ungerminated seeds, polyphenols are bound to non-starch polysaccharides in the cell walls. With the onset of germination, the carbohydrates are hydrolyzed by enzymes to provide sugars and energy for germination. The breakdown of non-starch polysaccharides releases polyphenols, which is reflected in the increase in total polyphenol content [26,28]. The increase in total polyphenol content, however, is not only due to the process of making existing polyphenols available but also their *de novo* synthesis [32].

Germination of chia seeds with 5, 20, 50, and 80% seawater, as well as germination with distilled and tap water, did not show statistically significant differences (*p* > 0.05). The statistical difference in germination with 10% seawater was significant (*p* < 0.05) compared to all samples except those germinated with 50 and 80% seawater and with distilled and tap water. Germination with 90% seawater showed statistically significant differences (*p* < 0.05) from every sample except those germinated with 20% seawater. Only samples germinated with 60, 70, and 100% seawater showed statistically significant differences (*p* < 0.05) from all other samples and from each other.

The highest measured value of total polyphenol content was 0.08 ± 0.00 mg of gallic acid/g of sample, observed in samples germinated with 100% seawater. A possible cause of the higher total polyphenol content could be the response of the germinating plant to stressful conditions induced by the higher concentration of NaCl [29]. Mane et al. described a positive correlation between total polyphenol content and water salinity [37].

## 4 Conclusion

The study revealed that chia seeds can germinate under various conditions, including distilled water, tap water, and low concentrations of seawater, as well as high concentrations up to 100% seawater. Antioxidant activity, measured by FRAP, DPPH, and ABTS methods, was generally higher in chia seeds germinated with distilled and tap water compared to ungerminated seeds, except for ABTS, where tap water germinated seeds showed increased activity. Germination in varying concentrations of seawater also increased antioxidant activity significantly compared to ungerminated seeds, with the highest observed in seeds germinated with 100% seawater, as confirmed by FRAP and DPPH methods. CUPRAC method demonstrated higher antioxidant reducing capacity in seeds germinated with distilled and tap water compared to ungerminated seeds, and all seawater concentrations showed increased reducing capacity, with the highest in seeds germinated with 100% seawater. Total polyphenol content, measured by the Folin–Ciocalteu method, increased significantly during germination across all samples, especially in seeds germinated with 100% seawater. In conclusion, germination significantly enhances antioxidant capacity and bioactive compound content in chia seeds, both under favorable and stressful saline conditions, with peak values observed in seeds germinated in 100% seawater. The results of our study clearly demonstrate that sprouting chia seeds under saline conditions – particularly in 100% seawater – significantly enhances their antioxidant capacity and polyphenol content. This offers an opportunity for the food industry to develop functional foods and ingredients with a higher amount of antioxidant compounds. Consequently, the gained results are promising both in terms of their potential nutritional benefits and their positive implications for environmental sustainability. However, the study has several limitations. It was conducted under controlled laboratory conditions, which may not fully reflect real-world agricultural or industrial environments. The study also focused solely on antioxidant properties and did not assess other potentially important factors such as germination rate under different salinities, sensory attributes of the sprouts, or long-term storage stability. Moreover, only one variety of chia seed was evaluated, and further research is needed to assess whether different genotypes exhibit similar responses to saline sprouting. Future studies, since more individual experiments will be needed, should investigate the scalability of this method and evaluate consumer acceptance of products developed using saline-sprouted seeds.
